# Synthesis, *In vitro* Antifungal and Antitumour Activity of Some Triorganotin(IV) N,C,N-Chelates

**DOI:** 10.1155/MBD.2002.91

**Published:** 2002

**Authors:** Libor Dostál, Aleš Růžička, Roman Jambor, Vladimír Buchta, Petra Kubanová, Jaroslav Holoček

**Affiliations:** 1 Department of General and Inorganic Chemistry Faculty of Chemical Technology University of Pardubice Studentská 95 Pardubice CZ-532 10 Czech Republic; 2 Department of Biological and Medical Science Charles Universily in Prague Facully of Pharmacy Heyrovského 1203 Hradec Králové CZ-500 05 Czech Republic

## Abstract

The *in vitro* antifungal activity of compounds **1**-**3** ($\left\{{\left[{\left({\text{CH}}_{3}\right)}_{2}{\text{NCH}}_{2}\right]}_{2}{\text{C}}_{6}{\text{H}}_{3}\right\}$ ${\text{R}}_{2}\text{SnX}$; (where X=Cl, R=*n*-Bu for **1**, X=Br, R=*n*-Bu for **2** and x=${\text{PF}}_{6}$, R=*n*=Bu for **3**)) was estimated with the help of a modified microdilution format of the M27-A guidelines and was compared with *in vitro* activity of their diphenyltin(IV) analogues **4** and **5** (where X=Br, R=Ph for **4** and X=${\text{PF}}_{6}$, R=Ph for **5**), and of drugs currently in clinical use (ketoconazole, fluconazole and amphotericin B). It was found that in coordinating solvents the more soluble derivative **2** is less active than the phenyl one (**4**), and compounds **1** and **3** are even inactive.

In this paper, the *in vitro* antitumour activity of ionic diphenyltin(IV) complexes **4** and **5** against seven tumoural cell lines of human origin is also reported. The preparation and characterization (H1, C13 and Sn119 NMR spectroscopy and electrospray ionization mass spectrometry) of the novel compound **3** is mentioned too.


### Synthesis, *in vitro* Antifungal and Antitumour Activity of Some Triorganotin(IV) N,C,N-Chelates

Libor Dostál[1,*], Aleš Růžička[1], Roman Jambor[1], Vladimír Buchta[2], Petra Kubanová[2] and Jaroslav Holoček[1]

[1]*Department of General and Inorganic Chemistry, Faculty of Chemical Technology, University of Pardubice, Studentská 95, CZ-532 10, Pardubice, Czech Republic.* [*]*E-mail: libor.dostal@upce.cz*
[2]*Department of Biological and Medical Science, Charles University in Prague, Faculty of Pharmacy, Heyrovského 1203, CZ-500 05, Hradec Králové, Czech Republic.*

### ABSTRACT

The *in vitro* antifungal activity of compounds **1-3** ({$[CH_3)_2NCH_2]_2C_6H_3$}$R_2SnX$; (where X=Cl, R=*n*-Bu for **1**, X=Br, R=*n*-Bu for **2** and x=$PF_6$, R=*n*=Bu for **3**)) was estimated with the help of a modified microdilution format of the M27-A guidelines and was compared with *in vitro* activity of their diphenyltin(IV) analogues **4** and **5** (where X=Br, R=Ph for **4** and X=$PF_6$, R=Ph for **5**), and of drugs currently in clinical use (ketoconazole, fluconazole and amphotericin B). It was found that in coordinating solvents the more soluble derivative **2** is less active than the phenyl one (**4**), and compounds **1** and **3** are even inactive.

In this paper, the *in vitro* antitumour activity of ionic diphenyltin(IV) complexes **4** and **5** against seven tumoural cell lines of human origin is also reported. The preparation and characterization ($^1H$, $^{13}C$ and $^{119}Sn$ NMR spectroscopy and electrospray ionization mass spectrometry) of the novel compound **3** is mentioned too.

### INTRODUCTION

Organotin(IV) compounds, especially triorganotin derivatives, have been extensively studied due to their potential *in vitro* antifungal activities against some medically important fungi /1/. Many compounds of this type display some *in vitro* antitumour activities against tumour cell lines of human origin /2,3/. Moreover, recently, Susperregui *et al.* have reported on *in vivo* trypanocidal activities of organotin(IV) compounds /4/.

In 1985 Atassi suggested that such *in vivo* antitumour activity and further clinical use of these compounds in medicine could be hampered by low water solubility /5/. A possibility to increase the water solubility of organotin compounds is to prepare compounds containing some heteroatoms in their structure /6/, or even ionic compounds. We have previously reported on such ionic compounds (water solubility ca. 200 mg/100 ml/room temperature) and their *in vitro* antifungal activities. Some of these organotin derivatives display MIC values (minimum inhibitory concentration $\mu mol \cdot l^{-1}$) comparable to those for currently used drugs /7/.

We now present the results of *in vitro* antitumour screening for two selected diphenyltin(IV) complexes **4** and **5** and also the *in vitro* antifungal activity of their dibutyltin(IV) analogues **1-3**.

### EXPERIMENTAL

### General comments and synthesis

All solvents were obtained from commercial sources, dried by standard procedures and distilled prior to use. Compounds **1**, **2**, **4** and **5** (see Fig. 1) were prepared according to literature procedure /7,8/.

[2,6-bis(dimethylaminomethyl)phenyl](di-*n*-butyl)stannylhexafluorophosphate 3: $AKPF_6$ (0.36 g, 1.94 mmol) was treated with a dichloromethane (200ml) solution of compound **2** (0.98 g, 1.94 mmol). The resulting suspension was stirred for 24 h at room temperature and then the insoluble material was filtered off, the dichloromethane solution was evaporated *in vacuo* and the residue was crystallized from a chloroform/*n*-pentane mixture (1:2), to give compound **3** as a pure white solid. Yield: 0.83 g (75%). M.p. 106-108°C. Anal. Calcd. for $C_{20}H_{37}N_2SnPF_6$: C 42.20; H 6.55; N 4.92. Anal. Found: C 42.03; H 6.52; N 5.01. $^{119}$Sn NMR ($CDCl_3$): δ (ppm) 53.6. $^{31}$P NMR ($CDCl_3$): δ (ppm) –145.32 (septet, $^1J(^{31}P, ^{19}F)$ = 713.4 Hz). $^{13}$C NMR ($CDCl_3$): δ (ppm) 133.89 (C(1), $^1J(^{119}Sn, ^{13}C)$ – not detected), 142.84 ©(2, 6), $^2J(^{119}Sn, ^{13}C)$ = 32.6 Hz), 126.25 (C(3,5), $^3J(^{119}Sn, ^{13}C)$ = 56.2 Hz), 131.66 (C(4), $^4J(^{119}Sn, ^{13}C)$ = 9.2 Hz), 64.91 ($NCH_2$, $^nJ(^{119}Sn, ^{13}C)$ = 29.1 Hz), 46.26 ($N(CH_3)_2$), 14.67 (C(1′), $^1J(^{119}Sn, ^{13}C)$ = 413.4 Hz), 28.11 (C(2′) $^2J(^{119}Sn, ^{13}C)$ = 29.1 Hz), 26.84 (C(3′), $^3J(^{119}Sn, ^{13}C)$ = 90.9 Hz), 13.24 (H(4′). $^1$H NMR ($CDCl_3$): δ (ppm) 7.44 (t, 1H, H(4)), 7.22 (d, 2H, H(3,5)), 3.80 (s, 4H, $NCH_2$), 2.58 (s, 12H, $N(CH_3)_2$, 1.57 (m, 4H, H(1′)), 1.42 (m, h*, H(2′, 3′), 0.90 (t, 6H, H (4′)), ESI-MS: MW = 570. Positive-ion MS: $[M-PF_6]^+$, m/z 425, 100%. Negative-ion MS: $[PF_6]^-$, m/z 145, 100%.

### Spectra

The solution state $^{119}$Sn, $^{31}$P, $^{13}$C and $^1$H NMR spectra were acquired at 134.28, 145.79, 90.56 and 360.13 MHz respectively, on a Bruker AMX 360 NMR spectrometer, using a 5 mm tuneable broad band probe at 300 K. Appropriate chemical shifts were calibrated on: $^1$H-residual peak of $CHCl_3$ (δ = 7.25 ppm), $^{13}$C-signal of $CDCl_3$ (δ = 77.0 ppm), $^{31}$P-external 85% $H_3PO_4$ (δ = 0.0 ppm) and $^{119}$Sn-external tetramethylstannane (δ = 0.0 ppm).

Electrospray ionisation (ESI) mass spectra were measured on an ion trap analyser (Esquire 3000, Bruker Daltonics) and on a quadrupole analyser (Platform, Micromass). The samples were dissolved in acetonitrile and analysed by direct infusion at a flow rate of 1 μl·min$^{-1}$. Mass spectra weree recorded in the range *m/z* 15-800, in both negative-ion and positive-ion mode.

### *In vitro* antifungal screening

The *in vitro* testing was carried out using a modified microdilution broth of the M27-A guidelines (National Committee for Clinical Laboratory Standards. Reference method for broth dilution antifungal susceptibility testing of yeasts. Approved standard. Document M27-A, Wayne, PA: National Committee for Clinical Laboratory Standards, 1997). Quality control strains (*Candida albicans* ATCC 90028, *Candida parapsilosis* ATCC 22019, *Candida krusei* ATCC 6258) and amphotericin B, fluconazole (Pfizer), ketoconazole (Janssen-Cilag, Beerse) as a reference drug were involved. All fungal strains were passaged on Sabouraud dextrose agar at 35°C prior to being tested.

The minimum inhibitory concentration (MIC) was determined by the following method: DMSO (dimethylsulfoxide) served as a diluent for all compounds tested. DMSO did not exceed a final concentration of 2%. RPMI 1640 (Sevapharma, Prague) medium, supplemented with L-glutamine and buffered with 0.165 M morpholinepropanesulfonic acid (Serva) to pH 7.0 (using 10 M NaOH), was used as a test medium. Each well of the microdilution tray was filled with 200 μl of the RPMI 1640 medium with a diluted compound, tested and then inoculated with 10 μl of suspension of a given fungal strain in sterile water. Fungal inoculum was prepared to give a final size of $5 \times 10^3 \pm 0.2$ CFU $ml^{-1}$. The trays were incubated at 35 °C and MICs read after 24 and 48h. Owing to slow growth, the *Trichophyton mentagrophytes* strain was read at 72 and 120 h. The MICs were determined visually and defined as 80% inhibition of the growth of control.

### *In vitro* antitumour screening

The protocol followed for the *in vitro* antitumour screenings has already been reported /9/.

### RESULTS AND DISCUSSION

### Characterization and structure

Compounds **1**, **2**, **4** and **5** were previously investigated /7,8,10,11/ by multinuclear NMR spectroscopy, electrospray ionisation mass spectrometry and X-ray diffraction techniques. The identity and purity of all derivatives were verified by elemental analysis too.

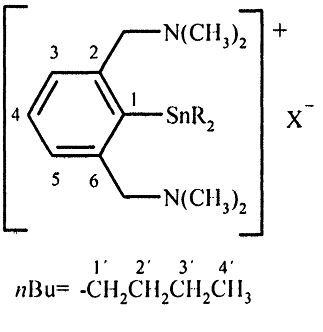

**1** R=*n*Bu, X=Cl

**2** R=*n*Bu, X=Br

**3** R=*n*Bu, X=$PF_6$

**4** R=Ph, X=Br

**5** R=Ph, X=$PF_6$

**Fig. 1:** Structure and numbering of compounds studied.

These compounds reveal the ionic structure with a five-coordinated tin atom and equivalent –$CH_2N(CH_3)_2$ groups (located in axial positions of a slightly distorted trigonal bipyramid) in solutions of coordinating solvents.

The compound **3** has been characterized with the help of NMR spectra parameters which correspond to appropriate ones in analogous above-mentioned compounds. One set of sharp signals was observed in the proton spectrum measured in $CDCl_3$ at room temperature, indicating equivalency of both amine donor and butyl groups, respectively. The $^{119}Sn$ chemical shift value (53.6 ppm) is comparable with previous results /8/ for ionic compounds (for example **1** and **2**), from the range for five-coordinated (or better [3+2] tin atom in ionic butyltin compounds. The extent of interatomic C(Bu)-Sn-C(Bu) angle can be calculated /12/ from the values of $^1J(^{119}Sn, ^{13}C)$ coupling constant (413.4 Hz, 116.1°). The symmetrical septet with $^1J(^{31}P, ^{19}F)$ = 713.4 Hz in the $^{31}P$ NMR spectrum shows that the $PF_6$ group which does not interact with the tin atom is present. On the basis of these findings, we can conclude that the vicinity of the tin central atom is slightly distorted trigonal bipyramidal, with donor amino groups in axial and carbon atoms in equatorial positions, forming the cationic unit. The hexafluorophosphate anion is out of the primary coordination sphere of the tin atom.

### *In vitro* antifungal activity

The results of *in vitro* antifungal screening for all compounds **1-5** are summarized in Table 1 together with MIC values (minimum inhibitory concentration μmol$^{-1}$) for conventional antimycotic drugs. The MICs for derivatives **4,5** were reported earlier elsewhere /7/ and are only used here for comparison of antimycotic activity of their di-*n*-butyl analogues **1-3**. The surprising discovery is that the bromobutyl derivative (**2**), more soluble in coordinating solvents, is less active than the phenyl one, and compounds **1** and **3** are even inactive.

**Table 1**

*In vitro* antifungal activity (MIC μmol l$^{-1}$) of compounds studied, determined by microdilution broth method

| Comp.[c] | MIC (μmol l$^{-1}$)[a, b] | | | | | | | |
|---|---|---|---|---|---|---|---|---|
| | TM | CA | CT | CK | CG | TB | AF | AC |
| | 72h/120h | 24h/48h | 24h/48h | 24h/48h | 24h/48h | 24h/48h | 24h/48h | 24h/48h |
| **1** | 125/250 | 500/>500 | 250/500 | >500/>500 | >500/>500 | >500/>500 | >500/>500 | 500/>500 |
| **2** | 31.25/62.5 | 7.8131.25 | 125/125 | 31.25/31.25 | 125/125 | 250/250 | 62.5/125 | 62.5 |
| **3** | 250/250 | 500/500 | 250/500 | >500/>500 | >500/>500 | >500/>500 | >500/>500 | 250/>250 |
| **4** | 1.95/1.95 | 15.6/125 | 250/250 | 7.81/15.6 | 125/250 | >250/250 | 7.81/15.6 | 7.81/15.6 |
| **5** | 1.95/1.95 | 7.81/15.6 | 62.5/>125 | 3.91/7.81 | 15.6/125 | >125/125 | 7.81/15.6 | 7.81/7.81 |
| [d] | 0.98/1.95 | 0.12/0.12 | 1.95/3.91 | 3.91/3.91 | 0.240.98 | 0.12/0.24 | 15.5/15.6 | 32.3/32.3 |
| [e] | 26.1/52.2 | 0.82/1.63 | 1.63/>417 | 52.2/105 | 13.1/52.2 | 3.26/6.53 | >417/417 | >417/417 |
| [f] | 2.16/21.6 | 1.08/2.16 | 2.16/4.33 | 2.16/4.33 | 2.16/2.16 | 0.27/0.27 | 0.27/0.54 | 0.54/2.16 |

[a] CA: *Candida albicans* ATCC 44895; TB: *Trichosporon beigelii* 1188; CT: *Candida tropicalis* 156; TM: *T. mentagrophytes* 445; CK: *Candida krusei* E28; AF: *Aspergillus fumigatus* 231; CG: *Candida glabrata* 20/I; AC: *Absidia corymbifera* 272.

[b] The limit of maximum concentration tested of given compounds was given with its solubility in DMSO.

[c] See Figure 1; [d] Ketoconazole; [e] Fluconazole; [f] Amphotericin B.

### *In vitro* antitumour activity

Compounds **4** and **5** were chosen for *in vitro* antitumour screening against seven tumoural cell lines of human origin: MFC-7 and EVSA-T: two breast cancers; WiDr: a colon carcinoma; IGROV: an ovarian cancer; M19MEL: a melanoma; A 498: a renal cancer and H 226: a non small cell lung cancer. The results of *in vitro* tests, given in the form of $ID_{50}$ (the inhibition doses ng · $ml^{-1}$) for these two derivatives are shown in Table 2 and compared with $ID_{50}$ values of some drugs with clinical applications. In spite of the fact that both derivatives **4** and **5** display increased water solubility and promising *in vitro* antifungal activity /7/, Table 2 shows that both tested compounds are entirely inactive on all tumour cell lines.

**Table 2**

*in vitro* inhibition doses $ID_{50}$ in ng · $ml^{-1}$ for compounds **4** and **5**.

| Compound[b] | $ID_{50}$ (ng · $ml^{-1}$)[a] | | | | | | |
|---|---|---|---|---|---|---|---|
| | MFC-7 | EVSA-T | WiDr | IGROV | M19MEL | A 498 | H 226 |
| **4** | 3460 | 3178 | 7538 | 8279 | 4420 | 7119 | 7903 |
| **5** | 5018 | 3301 | 8998 | 9328 | 5161 | 8879 | 8742 |
| cisplatin[c] | 699 | 422 | 967 | 169 | 558 | 2253 | 3269 |
| doxorubicine[c] | 10 | 8 | 11 | 60 | 16 | 90 | 199 |
| etoposide[c] | 2594 | 317 | 150 | 580 | 505 | 1314 | 3934 |

[a] MFC-7 and EVSA-T: two breast cancers; WiDr: a colon carcinoma; IGROV: an ovarian cancer; M19MEL: a melanoma; A 498: a renal cancer and H 226: a non small cell lung cancer.

[b] See Figure 1. [c] $ID_{50}$ values taken from the literature, see /3/.

### ACKNOWLEDGEMENTS

We are grateful to Prof. Marcel Gielen for mediation and Mr. R.G. Oostrum, Dr. J. Verweij, Prof. Dr. G. Stoter, Dr. K. Nooter (Laboratory of Experimental Chemotherapy and Pharmacology, Department of medical Oncology, Rotterdam Cancer Institute, NL-3008, The Netherlands) for performing the *in vitro* antitumour tests respectively. The authors thank the Grant Agency of the Czech Republic (grant No. 203/00/0920) and the Ministry of Education of the Czech Republic (grant MSM 111600002 – authors V.B. and P.K.) for financial support.

### REFERENCES

1. M. Nath, R. Yadav, G. Eng, P. Musingarimi, *Appl. Organomet. Chem.*, **8**, 29 (1999), and references cited therein.

2. M. Gielen, *Coord. Chem. Rev.*, **151**, 41 (1996).

3. M. Kemmer, M. Gielen, M. Biesemans, D. de Vos, R. Willem, *Metal Based Drugs*, **5**, 189 (1998).

4. J. Susperregui, M. Bayle, G. Lain, Ch. Giroud, T. Baltz, G. Déléris, *J. Med. Chem.*, **34**, 617 (1999).

5. G. Atassi, *Rev. Si, Ge, Sn, Pb Cpds.*, **8**, 219 (1985).

6. J. Susperregui, M. Bayle, J.M. Léger, G. Déléris, M. Biesemans, R. Willem, M. Kemmer, M. Gielen, *J. Organomet. Chem.*, **545-546**, 559 (1997).

7. A. Růžička, L. Dostál, R. Jambor, V. Buchta, J. Brus, I. Cisařová, M. Holčapek, J. Holoček, *Appl. Organomet. Chem.*, **16**, 315 (2002).

8. A. Růžička, R. Jambor, I. Cisařová, J. Holoček, *Chem. Eur. J.*, submitted to editor.

9. Y.P. Kepers, G.J. Peters, J. van Ark-Otte, B. Winograd, H.M. Pinedo, *Eur. J. Cancer*, **27**, 897 (1991).

10. R. Jambor, I. Cisařová, A. Růžička, J. Holoček, *Acta Crystallogr.*, **C57**, 373 (2001).

11. J. Holoček, A. Lyčka, *Inorg. Chim. Acta*, **118**, L15 (1986).

